# HIV incidence from the first population-based cohort study in India

**DOI:** 10.1186/1471-2334-13-327

**Published:** 2013-07-17

**Authors:** Lalit Dandona, G Anil Kumar, Vemu Lakshmi, G Md Mushtaq Ahmed, Mohammed Akbar, Sri P Ramgopal, Talasila Sudha, Michel Alary, Rakhi Dandona

**Affiliations:** 1 Public Health Foundation of India, New Delhi, India; 2 Institute for Health Metrics and Evaluation, University of Washington, Seattle, WA, USA; 3 Department of Microbiology, Nizam’s Institute of Medical Sciences, Hyderabad, India; 4 URESP, Centre de recherche FQRS du CHA universitaire de Québec, Québec, Canada

**Keywords:** HIV incidence, India, Population-based cohort, Rural, Spouse

## Abstract

**Background:**

Understanding about who acquires new HIV infection and the determinants of why some persons get infected and others do not is fundamental to controlling HIV in the population. We assess HIV incidence and its associations in the population of a high HIV burden district in Andhra Pradesh state in southern India by a population-based longitudinal cohort study.

**Methods:**

We re-surveyed a population-based cohort of 12,617 adults in Guntur district of Andhra Pradesh for which we had reported a baseline HIV prevalence of 1.72% (rural 1.64%, urban 1.89%) among the 15–49 years age group in 2004–2005. We conducted interviews to assess risk behaviour and performed HIV testing again in 2010–2011. We assessed the rate of new HIV infection and its associations using multiple logistic regression.

**Results:**

The participation rate in the follow-up was 74.9% and 63.9% of the baseline rural and urban samples, respectively. Over a mean follow-up of 5.63 years, the incidence of HIV was 1.26 per 1000 person-years (95% CI 0.83-1.69), after adjusting for slight compositional bias in the follow-up sample. The incidence per 1000 person-years was higher among rural men (1.68) than urban men (0.85), and among rural women (1.28) than urban women (0.54). The strongest association with incidence was a HIV positive spouse in the baseline for both men (odds ratio 266, 95% CI 62–1137) and women (odds ratio 28, 95% CI 9–88). Among men the other significant associations with HIV incidence were frequent use of condom for sex over the past 6 months, non-circumcision, more than one lifetime woman sex partner or ever visited sex worker, and transport-related occupation; for women the other significant associations were having had HIV testing other than antenatal check-up, previously married but currently not, and tobacco use.

**Conclusion:**

These first population-based cohort incidence data from India suggest that rural areas of high HIV burden states would need more attention to prevent new HIV infections, and that spouses of HIV positive persons and some other risk groups need to be targeted more effectively by HIV prevention programmes.

## Background

Understanding about who acquires new HIV infection and the determinants of why some persons get infected and others do not is fundamental to controlling HIV in the population. Population-based longitudinal studies of new HIV infection, or incidence, have been reported so far only from sub-Saharan Africa [1-12]. Reliable estimation of HIV incidence requires an adequate number of new HIV infections over the follow-up period, which can be difficult to achieve in places that have a relatively lower level of HIV infection in the population. Other methods of estimating HIV incidence include assessing changes in prevalence over time and use of laboratory tests that may detect recent HIV infections [13,14]. However, both these approaches have significant limitations, and the population-based longitudinal approach remains the standard to measure HIV incidence because this enables direct assessment of HIV seroconversion in individuals who are HIV negative at baseline.

In 2006, the burden of HIV in India was estimated to be 5.7 million persons, but population-based studies revealed that this was being overestimated and that the actual burden was closer to half of this [15]. Even with this downward revision, UNAIDS still estimates India to have one of the largest HIV burden in the world with 2.4 million persons with HIV in 2009 [16]. According to the National AIDS Control Organization of India the prevalence of HIV among adults was estimated to be 0.31% in 2009 [17]. Among the large states of India, this prevalence was estimated to be highest in Andhra Pradesh (0.90%) followed by Karnataka (0.63%), both states in southern India. Among the small north-eastern states of India were the epidemiology of HIV is heavily influenced by intravenous drug use, Manipur was estimated to have the highest adult HIV prevalence (1.40%) followed by Nagaland (0.78%). While the prevalence of HIV in the population has been better understood in India over the past few years through population-based studies [18-21], so far there have been no data reported on the rate of new HIV infections, i.e. incidence of HIV, in the population from a longitudinally followed-up population-based cohort. In this paper we report HIV incidence from such a cohort in a high HIV burden district in the south Indian state of Andhra Pradesh followed up for over 5 years, and the factors associated with HIV incidence.

## Methods

### Population sample

We conducted a cross-sectional population-based survey of HIV in Guntur district of Andhra Pradesh, India in 2004–2005, which contributed to revision of the method for estimating the HIV burden in India [18]. Guntur district had a population of 4.46 million in the 2001 census and 4.89 million in the 2011 census [22,23]. Details of the sampling have been published previously [18]. Briefly, this study was conducted in 32 rural and 34 urban clusters, including 2 urban homeless clusters; selected using a stratified random method to represent the adult population of Guntur district and systematic sampling was done to select households in order to get 200–230 eligible persons 15–49 years old in each cluster. Of the eligible 13,838 people 15–49 years of age, the participation rate was 12,617 (91.2%) for HIV prevalence estimation in the 2004–2005 survey. These 12,617 persons included 60 homeless persons in the sample. We repeated the survey in this population-based cohort in 2010–2011 to estimate the population level incidence of HIV and its associations.

**Figure 1:**
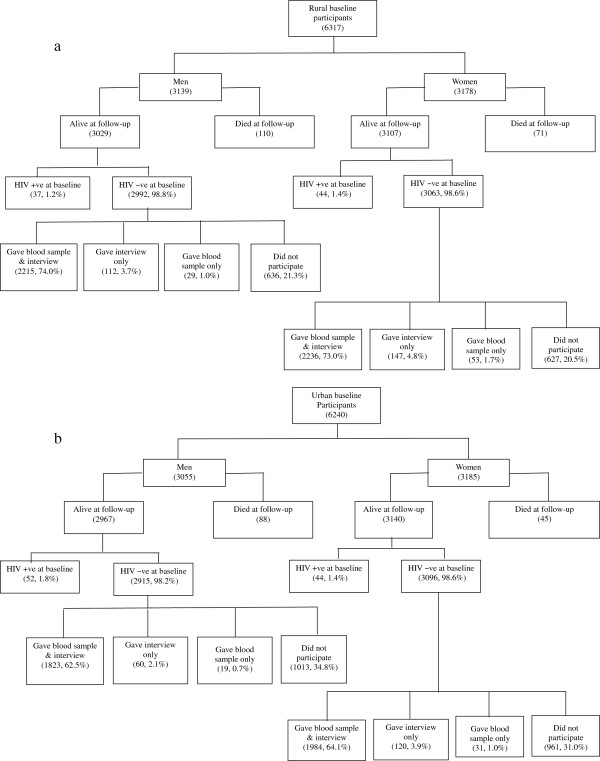
**Participation profile in follow-up survey. a**. rural. **b**. urban.

Of the 12,557 persons in the 2004–2005 baseline study, excluding the 60 homeless persons as there was no way to trace them, 7,222 (57.5%) were living in the same residence during the follow-up period up to 2010–2011 (which included 229 who had died) and 5,335 (42.5%) had moved from their original residence (Figure 1a and 1b). Of these 5,335 persons, 3,562 (66.8%) could be traced in other locations including 75 who had died. Using age-sex-specific death rates based on the total 304 known deaths in the baseline sample and the death rate among those who were HIV positive at baseline, we estimated that 10 of the 1,773 untraceable persons would have died including 2 who were HIV positive at baseline, resulting in an estimated 314 deaths among the baseline sample excluding the homeless. These deaths included 52 among the 229 persons who were HIV positive at baseline, excluding the homeless. This gave an eligible sample of 12,066 persons for the HIV incidence estimation who in the baseline study were 15–49 years of age and HIV negative, and were alive at follow-up.

### Procedures

Data were collected from May 2010 to July 2011, using procedures standardized in the baseline study [18], and refined further to meet the data requirements of the follow-up study. In the baseline study we assigned a specific identification number to each participant. The personal identifies were delinked from the identification number during analysis. At the time of the follow-up study, the name, address, age, religion and caste of each participant was extracted from the database to trace them. Trained field staff used this information to identify these persons for follow-up and obtained written informed consent from them for participation. At least five attempts were made to reach all eligible people, including those who had migrated. The field staff conducted confidential interviews of participants in a private setting inside or near the house of the respondent to get information about their socio-demographic background, sexual and other behaviour that may be associated with incidence of HIV and the utilization of HIV interventions as relevant. The interviewers did not have any knowledge of the data of the respondents from the baseline survey. The instruments (questionnaires) were bilingual in the local language Telugu and English.

A blood sample from each respondent was collected on a filter paper (Whatman No. 3; Whatman International Ltd, Maidstone, Kent, UK) by finger-prick method, preferably six drops which was allowed to dry and then appropriately labelled and sealed in zip-lock polythene bags and were transported weekly to the laboratory in Hyderabad. Field staff assessed the haemoglobin level by comparing the colour of the blood sample on filter paper with the Haemoglobincolour scale developed by the World Health Organization [24]. If the haemoglobin level was 6 gm/dl or less, the respondents were considered as severely anaemic, were given iron and folic tablets and advised to visit the doctor immediately for further assessment. If the haemoglobin level was 7–10 gm/dl, the respondent was considered as anaemic, and was advised that blood iron level can be improved by eating more green leafy vegetables and were given iron and folic tablets, and advised to see a doctor for follow-up. If the haemoglobin level was 11 gm/dl or more, the respondent was advised that eating green leafy vegetables would facilitate in maintaining the haemoglobin level and were given multi-vitamin tablets. No monetary incentive was given to the study participants.

Standardized laboratory methods were used for HIV testing. Briefly, the dried blood spots were transported from the field to the laboratory every week, where they were stored at 2–8°Celsius and tested for HIV. Most of the samples were tested for HIV within two weeks of collection, and all within two months. First a fourth-generation immunoassay (Genscreen Ultra HIV Ag/Ab, Biorad Laboratories, USA) was used, and the positive samples were tested with another type of fourth-generation immunoassay (Vidas HIV Duo Ultra, bio*Mérieux*, *Marcy-l’Etoile*, France) to confirm the presence of HIV antibody or antigen. Those positive for antibodies were re-tested with a third-generation rapid HIV test (HIV Tridot; J. Mitra, New Delhi, India) to distinguish between HIV-1 and HIV-2 antibodies. Quality assurance testing was performed on 10% of the samples negative for HIV antibody or antigen by repeating the fourth-generation immunoassay (Biorad). All of these samples remained negative on repeat testing.

Because HIV test results would not be linked with participant identifiers openly, HIV test results for each participant were sent to the nearest public sector integrated counselling and testing centre (ICTC) using a unique identification number for each participant. The study participants were given their unique identification number and advised to go to the respective ICTCs within a specified time period. Those who had tested positive for HIV in the study were tested again at the ICTC and given the final HIV test result, counselling and treatment as appropriate.

Ethics approval for this study was obtained from the institutional ethics committees of the Public Health Foundation of India, New Delhi, India, Nizam’s Institute of Medical Sciences, Hyderabad, India, and CH*A*universitaire de Québec, Québec, Canada. Follow-up data collection was approved, with the interviewers not having any knowledge of the respondent data on their behaviour and HIV status from the baseline survey. No study staff collecting data in the follow-up study had access to individual level information from the baseline study about the sexual behaviour reported by the respondents or their HIV/STI status.

### Statistical analysis

Data were entered in a MS Access database designed for this study by data entry operators, which were scrutinized to detect and correct errors. Data were analysed using SPSS software Version 17 (IBM SPSS statistics standard, USA).

New HIV infections detected at follow-up in persons who were HIV negative in the baseline study were considered for calculating the incidence. The follow-up period for each person was considered to calculate the total person-years needed as the denominator for calculating the HIV incidence. HIV incidence in each cluster was age-standardized separately for men and women with the last available age distribution in the rural and urban populations of Guntur district from the 2001 census. Relevant weights were used for aggregate rural, urban and total estimates, as described in detail earlier for the baseline study [18]. HIV incidence was estimated by dividing the new HIV cases during the follow-up period by the number of person-years at risk during this period. The 95% confidence intervals of these HIV incidence estimates were calculated taking into account the design effect of the cluster sampling strategy [25].

We assessed the compositional bias of the follow-up sample as compared with the baseline sample based on the behavioural risk factors associated with HIV in the baseline study, which included no circumcision, multiple sex partners or visiting sex workers, sex after consuming alcohol, tattooing, recreational drugs, men having sex with men (MSM), and having had blood transfusion [26]. We applied the relative impact of each of these risk factors, as calculated earlier [26], to the distribution of these risk factors in the baseline HIV negative participants and to the distribution of these risk factors in the follow-up participants, and aggregated the impact for the various risk factors among rural men, urban men, rural women and urban women to assess if the HIV incidence may have been over- or under-represented in these groups due to the potential composition bias related to these risk factors. We adjusted the estimates of HIV incidence accordingly.

As the homeless in the baseline sample could not be followed up, we estimated the HIV incidence among homeless men and women by applying the ratio of the incidence to baseline HIV prevalence among the non-homeless to the baseline HIV prevalence among homeless men (19.5%) and women (21.1%), assuming that this ratio may be similar. We then used this incidence estimate among the homeless to adjust the urban and overall incidence among men and women.

We had only one follow-up time point in this study and the duration of follow-up was similar among the study participants. We used multiple logistic regression models to assess the association of socio-economic variables and behavioural variables with HIV incidence separately first, and then in a combined model with all variables. We performed these analyses separately for men and women. We started with risk variables assessed for association with prevalence [26], excluding some of those that had insufficient variability in incidence between the sub-groups. Besides this, we added some new behavioural variables in the model for which data were available, which included spouse HIV status at baseline, injections received in the last 12 months, travel outside place of residence, residence relocation, HIV testing, and contact with any HIV prevention programme.

## Results

### Participation rate

Of the total sample of 12,066 persons eligible for HIV incidence estimation, 8,390 (69.5%) gave blood sample in the follow-up study for HIV testing. This included 3,857 (46%) urban residents and 4,304 (51.3%) women. The participation profile for the rural and urban residents is shown in Figure 1a and 1b. Among the eligible sample from the baseline study, the participation rate for HIV testing in the follow-up study was 75% for both rural men and woman, and was 65% for urban women and 63.1% for urban men. The mean follow-up duration was 5.63 years (range 5.17-6.33 years) and the total follow-up 47,236 person-years. The mean follow-up duration was the same for rural and urban men (5.64 and 5.65 years, respectively) as well as women (5.64 and 5.61 years, respectively).

### Compositional bias

Table 1 shows the compositional bias of the follow-up sample based on the behavioural risk factors associated with HIV in the baseline study. Based on the distribution of these behavioural risk factors at baseline among those who participated in the follow-up and those who did not, excluding the homeless, we found that the HIV incidence estimation was under-estimated by 4.3% in the non-homeless urban men, was over-estimated by 4.5% in the non-homeless urban women, over-estimated by 5.4% in rural men and under-estimated by 9.3% in rural women. Applying the incidence to prevalence ratio of non-homeless urban men and women to the homeless urban men and women, we estimated that due to the absence of follow-up of the homeless sample from baseline, HIV incidence was underestimated by 9.8% in urban men and by 5.6% in urban women.

**Table 1:** Compositional bias of the follow-up sample based on the behavioural risk factors associated with HIV in the baseline study

		**Proportion having this risk factor**			**Relative impact on HIV estimation**^**b**^		
**Variables**	**Baseline HIV prevalence**	**Among those who did not participate in follow-up**	**Among those who participated in follow-up**	**Relative impact of risk factor on HIV per unit population**^**a**^	**Among those who did not participate in follow-up**	**Among those who participated in follow-up**	**Ratio of participated to not participated**^**c**^
**Urban men (Number not participated, number participated)**							
Not circumcised (831, 2227)	3.95	0.806	0.725	1.00	3.184	2.864	
More than one lifetime woman sex partners or ever visited sex worker (1127, 1815)	4.23	0.433	0.440	0.76	1.392	1.413	
More than two lifetime women sex partners or ever visited sex worker (1131, 1817)	4.49	0.333	0.341	0.58	0.868	0.887	
Have sex after consuming alcohol (1128, 1816)	5.41	0.234	0.230	0.58	0.734	0.721	
Had tattooing (1145, 1841)	7.45	0.076	0.088	0.27	0.153	0.177	
Used any recreational drugs (1131, 1798)	14.29	0.016	0.010	0.13	0.030	0.019	
Men who had sex with men (830, 2226)	11.25	0.020	0.023	0.13	0.029	0.034	
Had blood transfusion (1145, 1841)	8.08	0.029	0.029	0.10	0.023	0.023	
***Overall urban men***	***2.45***				***6.413***	***6.138***	***0.957***
**Rural men (Number not participated, number participated)**							
More than two lifetime women sex partners or ever visited sex worker (830, 2226)	2.83	0.380	0.412	0.44	0.473	0.514	
More than one lifetime woman sex partner or ever visited sex worker (830, 2226)	2.28	0.525	0.574	0.40	0.479	0.523	
Have sex after consuming alcohol (830; 2,225)	3.20	0.246	0.235	0.28	0.220	0.211	
Not circumcised (1131, 1816)	1.97	0.915	0.909	0.13	0.234	0.233	
Had tattooing (838, 2243)	2.99	0.053	0.067	0.05	0.008	0.010	
Had blood transfusion (838, 2242)	3.51	0.013	0.020	0.02	0.001	0.001	
Used any recreational drugs (831, 2227)	3.00	0.011	0.010	0.01	0.000	0.000	
Men who had sex with men (1128, 1815)	2.00	0.012	0.018	0.00	0.000	0.000	
***Overall rural men***	***1.72***				***1.415***	***1.492***	***1.054***
**Urban women (Number not participated, number participated)**							
More than one lifetime man sex partners (1058, 1941)	8.33	0.050	0.052	0.21	0.087	0.091	
Blood transfusion done (838, 2288)	2.08	0.074	0.099	0.01	0.002	0.002	
***Overall urban women***	***1.90***				***0.089***	***0.093***	***1.045***
**Rural women (Number not participated, number participated)**							
More than one lifetime man sex partners (826, 2270)	3.00	0.126	0.113	0.21	0.079	0.071	
Blood transfusion done (1113, 2015)	3.20	0.054	0.073	0.01	0.002	0.002	
***Overall rural women***	***1.57***				***0.081***	***0.074***	***0.907***

^a^Relative impact of risk factors on HIV per unit population were published earlier from this study; these are based on computation of population impact number using the relative risk of HIV associated with that factor and the prevalence of that factor [26].

^b^Relative impact on HIV estimation calculated by multiplying these three separately for those who participated and those who did not participate in the follow-up: HIV prevalence in those having that factor, proportion having this factor among those who participated/did not participate, and relative impact of that factor on HIV in a unit population.

^c^Ratio for each group (urban men, rural men, urban women, rural women) calculated by summing up the relative impact on HIV estimation for all factors in each group separately for those who participated and those who did not participate and divided the former number with the later. This ratio would give the over- or under-estimation of HIV in each group from those who participated in the follow-up based on the distribution of the risk factors in those who participated and those who did not.

### Incidence

Of the 8,390 persons who were HIV negative at baseline and gave blood sample in the follow-up study, 48 had seroconverted at follow-up, which included 46 (95.8%) HIV-1, 2 (4.2%) HIV-2, and none who were only antigen positive. The overall incidence of HIV among persons 15–49 years of age at baseline, adjusted for age, sex and rural/urban distribution of the population in Guntur district and adjusted for the compositional bias as described above, was 1.25 per 1000 person-years (95% CI 0.82-1.68) (Table 2). Based on the rural/urban residence at baseline, the incidence was two times higher among rural men than urban men and over two times higher among rural women than urban women, resulting in a higher HIV incidence among rural adults (1.47 per 1000 person-years; 95% CI 0.75-2.19) than in urban adults (0.70 per 1000 person-years; 95% CI 0.32-1.07). The incidence was slightly higher among men than among women in both rural and urban areas.

**Table 2:** Population-based incidence of HIV in Guntur district, Andhra Pradesh, India

	**Men**		**Women**		**Total**	
	**Adjusted HIV incidence**^**a **^**(Per 1000 person-years)**	**95% confidence interval (design effect)**	**Adjusted HIV incidence**^**a **^**(Per 1000 person-years)**	**95% confidence interval (design effect)**	**Adjusted HIV incidence**^**a **^**(Per 1000 person-years)**	**95% confidence interval (design effect)**
Rural	1.66	0.56-2.77 (2.39)	1.27	0.44- 2.10 (1.80)	1.47	0.75-2.19 (2.34)
Urban	0.85	0.29-1.41 (1.00)	0.54	0.10-0.98 (1.04)	0.70	0.32-1.07 (1.14)
Total	1.43	0.80-2.07 (1.67)	1.07	0.56-1.57 (1.49)	1.25	0.82-1.68 (1.84)

^a^Adjustments explained in the methods section.

Of the 8,390 persons participating in the follow-up, 2,827 (33.7%) had moved residence to another location. There was no difference in HIV incidence among those who had moved (16 of 2827, 0.57%) and those who had not moved (32 of 5563, 0.58%) during the follow-up period. There was no major difference in the magnitude of HIV incidence between the rural-to-urban and urban-to-rural relocations though the numbers were small in these two groups (Table 3). If the rural/urban residence at follow-up was considered instead of the residence at baseline, overall the magnitude of rural HIV incidence was still about twice the urban incidence. There is a suggestion of higher incidence rate among men who relocated from rural to urban residence, and among women who relocated from urban to rural residence, though the small numbers do not allow firm comparisons.

**Table 3:** New HIV among participants who relocated residence between the baseline and follow-up studies

**Relocation**	**Number**	**Number (%) who acquired HIV**
**Rural to rural**		
Men	370	2 (0.54)
Women	416	2 (0.48)
*Total*	*786*	*4 (0.51)*
**Rural to urban**		
Men	75	2 (2.67)
Women	83	0
*Total*	*158*	*2 (1.27)*
**Urban to rural**		
Men	36	0
Women	69	1 (1.45)
*Total*	*105*	*1 (0.95)*
**Urban to urban**		
Men	851	3 (0.35)
Women	927	6 (0.65)
*Total*	*1,778*	*9 (0.51)*

As compared with the baseline HIV adult prevalence of 1.72% (rural 1.64%, urban 1.89%) [18], the incidence rate per 100 person-years was 7.3% of the baseline prevalence. This rate was higher for rural adults (9.0%) than for urban adults (3.7%).

### Factors associated with HIV incidence

Table 4 shows the associations of HIV incidence with socio-demographic and behavioural variables for men using multiple logistic regression. The strongest association was with having a HIV-positive spouse at baseline (odds ratio 412, 95% CI 84–2020). The other significant associations were with use of condom often or always for sex over the past 6 months (odds ratio 14.5), no circumcision (odds ratio 13.8), more than one lifetime women sex partner or ever visited sex worker (odds ratio 3.5), and transport-related occupation (odds ratio 3.1). The R^2^ for the final logistic regression model including both the socio-demographic and behavioural variables was 0.34.

**Table 4:** Association of socio-demographic and behavioural variables with acquiring HIV among men during the follow-up period using multiple logistic regression

**Variable**^**a**^	**Category**	**Number (% of total)**	**Number (%) who acquired HIV**	**Adjusted odds**^**b **^**of acquiring HIV (95% CI)**	**Adjusted odds**^**c **^**of acquiring HIV (95% CI)**
***Socio-demographic***					
Age (years)	20-29	1272 (31.1)	6 (0.47)	1.0	
	30-39	1200 (29.4)	5 (0.42)	1.0 (0.3–4.0)	
	40-49	983 (24.1)	7 (0.71)	1.6 (0.4–5.9)	
	50-55	631 (15.4)	5 (0.79)	2.3 (0.6–9.4)	
Education	Schooling	3078 (76.2)	15 (0.49)	1.0	
	No Schooling	960 (23.8)	8 (0.83)	1.1 (0.4–2.9)	
Marital status	Never married	511 (12.7)	3 (0.59)	1.0	
	Currently married/Cohabiting	3448 (85.4)	16 (0.46)	0.5 (0.1–2.1)	
	Previously married	78 (1.9)	4 (5.13)	3.2 (0.5–21.1)	
Standard of living index^d^	Quartile IV	1051 (25.8)	2 (0.19)	1.0	1.0
	Quartile III	1051 (25.8)	4 (0.38)	2.0 (0.4–11.2)	1.7 (0.3–10.9)
	Quartile II	1046 (25.6)	5 (0.48)	2.5 (0.5–13.5)	2.2 (0.4–13.5)
	Quartile I	930 (22.8)	12 (1.29)	5.9 (1.2–29.5)	4.5 (0.9–23.5)
Place of residence at baseline	Rural	2244 (54.9)	14 (0.62)	1.0	
	Urban	1842 (45.1)	9 (0.49)	1.1 (0.4–2.9)	
Residence relocation	Rural to urban	75 (1.8)	2 (2.7)	3.9 (0.7–20.5)	
	All others	4011 (98.2)	21 (0.5)	1.0	
Occupation	Other than the categories below	3170 (78.5)	15 (0.47)	1.0	1.0
	Transport related	355 (8.8)	5 (1.41)	3.0 (1.0–8.3)	3.1 (1.0–9.7)
	Unskilled labour	512 (12.7)	3 (0.59)	1.0 (0.3–3.7)	0.9 (0.2–3.6)
***Behavioural***					
Spouse HIV status at baseline	HIV –ve	2810 (68.8)	7 (0.25)	1.0	1.0
	HIV + ve	17 (0.4)	6 (35.29)	411.9 (84.0–2019.6)	266.5 (62.5–1136.7)
	Never married at baseline	1089 (26.7)	5 (0.46)	1.5 (0.4–6.1)	1.3 (0.3–5.0)
	Spouse HIV status not available at baseline	170 (4.2)	5 (2.94)	9.5 (2.6–34.5)	7.4 (2.0–26.8)
Circumcision	Yes	744 (18.6)	1 (0.13)	1.0	1.0
	No	3265 (81.4)	22 (0.67)	13.4 (1.0–181.4)	13.8 (1.2–153.5)
Women sex partner	Never had sex or only one lifetime women partner, never visited sex worker	2301 (60.8)	6 (0.26)	1.0	1.0
	More than one lifetime women partner or visited sex worker	1483 (39.2)	16 (1.08)	3.2 (1.0–10.1)	3.8 (1.3–11.5)
Used condom for sex in last 6 months	Not used/Do not remember	3143 (82.7)	14 (0.45)	1.0	1.0
	Never had sex/No sex in last 6 months	563 (14.8)	4 (0.71)	2.6 (0.7–10.1)	2.3 (0.6–8.7)
	Half of the time/rarely	34 (0.9)	1 (2.94)	4.0 (0.4–41.1)	3.5 (0.3–36.6)
	Often/always	60 (1.6)	3 (5.00)	17.8 (3.7–85.9)	14.5 (3.2–65.4)
Have sex after consumption of alcohol	Never had sex/Do not drink alcohol	1620 (42.7)	4 (0.25)	1.0	
	Usually	217 (5.7)	2 (0.92)	1.5 (0.1–15.0)	
	Some time	904 (23.8)	8 (0.88)	3.0 (0.7–13.6)	
	Never	1057 (27.8)	8 (0.76)	1.8 (0.4–7.7)	
Had blood transfusion	Never	3901 (97.3)	21 (0.54)	1.0	
	Ever	109 (2.7)	2 (1.83)	5.0 (0.9–27.9)	
Had tattooing	No	3633 (90.6)	20 (0.55)	1.0	
	Yes	376 (9.4)	3 (0.80)	0.9 (0.2–3.8)	
Smoking or chewing tobacco	Never	1612 (40.1)	3 (0.19)	1.0	
	Ever	2404 (59.9)	20 (0.83)	4.0 (0.8–20.5)	
Injections received in the last 12 months	No	1713 (42.7)	7 (0.41)	1.0	
	Yes	2301 (57.3)	16 (0.70)	1.4 (0.5–3.8)	
Travel outside place of residence	Never	299 (7.4)	1 (0.33)	1.0	
	Daily	1144 (28.4)	9 (0.79)	2.4 (0.3–22.6)	
	Weekly	748 (18.6)	2 (0.27)	0.6 (0–8.3)	
	Monthly	749 (18.6)	6 (0.80)	2.2 (0.2–22.2)	
	Once in a while	1085 (27.0)	5. (0.46)	1.6 (0.2–16.6)	
HIV testing	No	2840 (72.5)	15 (0.53)	1.0	
	Yes	1077 (27.5)	8 (0.74)	1.1 (0.4–3.0)	

^a^All variables based on status as at follow-up, except rural or urban residence and spouse HIV status. Total number of men in the socio-demographic only model was 4,030, in the behavioural only model was 3,757, and the combined model was 3,773 because of missing data for these variables: marital status for 49, education status for 48, occupation status for 49, Standard of living index for 8, sex with women for 302, Used condom for sex in last 6 months for 286, have sex after consumption of alcohol for 288, circumcision for 77, had tattooing for 77, had blood transfusion for 76, smoking or chewing tobacco for 70, HIV testing other than ANC for 169, injections received in the last 12 months for 72, travel outside place of residence for 61.

^b^Adjusted odds based on separate multiple logistic regression models for socio-demographic variables and for behavioural variables.

^c^Adjusted odds based on a single multiple logistic regression model that included socio-demographic and behavioural variables that were significant in the separate models.

^d^Standard of living index based on living conditions and ownership of assets, as used in National Family Health Survey-2 [27]; quartiles defined according to the baseline study distribution [18].

For women too, in the socio-demographic and behavioural multiple logistic regression model, the strongest association of HIV incidence was with having a HIV-positive spouse at baseline (odds ratio 27.7, 95% CI 8.7-88.3) (Table 5). The other significant associations were with having had HIV testing other than related to antenatal check-up (odds ratio 4.0), having been married previously but not currently (odds ratio 3.7), and tobacco smoking or chewing (odds ratio 3.3). The R^2^ for the final logistic regression model including both the socio-demographic and behavioural variables for women was 0.24.

**Table 5:** Association of socio-demographic and behavioural variables with acquiring HIV among women during the follow-up period using multiple logistic regression

**Variable**^**a**^	**Category**	**Number (% of total)**	**Number (%) who acquired HIV**	**Adjusted odds**^**b **^**of acquiring HIV (95% CI)**	**Adjusted odds**^**c **^**of acquiring HIV (95% CI)**
***Socio-demographic***					
Age (years)	20-29	1317 (30.6)	5 (0.38)	1.0	
	30-39	1342 (31.2)	12 (0.89)	2.2 (0.8–6.5)	
	40-49	1168 (27.1)	5 (0.43)	0.9 (0.2–3.4)	
	50-55	477 (11.1)	3 (0.63)	1.0 (0.2–4.5)	
Education	Schooling	2390 (56.6)	12 (0.50)	1.0	
	No Schooling	1829 (43.4)	13 (0.71)	0.7 (0.3–1.7)	
Marital status	Never married / Currently married / Cohabiting	3704 (87.8)	14 (0.38)	1.0	1.0
	Previously married	515 (12.2)	11 (2.14)	5.3 (2.2–12.6)	3.7 (1.3–10.2)
Standard of living index^d^	Quartile IV	1034 (24.1)	1 (0.10)	1.0	1.0
	Quartile III	1099 (25.6)	3 (0.27)	2.6 (0.3–25.2)	2.4 (0.2–24.0)
	Quartile II	1106 (25.8)	10 (0.90)	7.6 (0.9–62.0))	7.1 (0.9–57.0)
	Quartile I	1053 (24.5)	11 (1.04)	7.9 (0.9–65.7)	6.5 (0.8–52.6)
Place of residence at baseline	Rural	2289 (53.2)	16 (0.70)	1.0	
	Urban	2015 (46.8)	9 (0.45)	0.6 (0.3–1.6)	
Residence relocation	Urban to rural	69 (1.6)	1 (1.5)	2.4 (0.3–20.4)	
	All others	4011 (98.4)	24 (0.6)	1.0	
Occupation	Other than the categories below	3858 (91.5)	21 (0.54)	1.0	
	Involving regular mobility	49 (1.2)	1 (2.04)	2.5 (0.3–19.7)	
	Unskilled labour	310 (7.4)	3 (0.97)	1.3 (0.3-4.8)	
***Behavioural***					
Spouse HIV status at baseline	HIV –ve	2983 (69.3)	9 (0.30)	1.0	1.0
	HIV + ve	48 (1.1)	7 (14.58)	45.9 (14.3–147.3)	27.7 (8.7–88.3)
	Never married at baseline	367 (8.5)	1 (0.27)	1.3 (0.2–10.4)	1.1 (0.1–9.4)
	Spouse HIV status not available at baseline	906 (21.1)	8 (0.88)	3.3 (1.1–7.8)	1.4 (0.4–4.4)
Men sex partners	Never had sex or only one lifetime partner	3613 (96.2)	21 (0.58)	1.0	
	More than one lifetime men partner	142 (3.8)	3 (2.11)	2.2 (0.6–8.3)	
Had blood transfusion	Never	3727 (88.9)	21 (0.56)	1.0	
	Ever	465 (11.1)	4 (0.86)	1.2 (0.4–3.8)	
Had tattooing	No	3559 (84.9)	19 (0.53)	1.0	
	Yes	633 (15.1)	6 (0.95)	1.1 (0.4–3.2)	
Smoking or chewing tobacco	Never	4039 (96.3)	21 (0.52)	1.0	1.0
	Ever	157 (3.7)	4 (2.55)	4.4 (1.2–15.6)	3.3 (1.0–11.3)
Injections received in the last 12 months	No	1802 (43.0)	8 (0.44)	1.0	
	Yes	2391 (57.0)	17 (0.71)	1.0 (0.4–2.6)	
Travel outside place of residence	Never	509 (12.1)	6 (1.18)	1.0	
	Daily	227 (5.4)	1(0.44)	0.3 (0–2.8)	
	Weekly	84 (2.0)	1 (1.19)	0.5 (0–4.7)	
	Monthly	349 (8.3)	3 (0.86)	0.4 (0.1–1.9)	
	Once in a while	3046 (72.3)	14 (0.46)	0.3 (0.1–0.9)	
Contacted by any one for HIV prevention programme	No	3479 (86.5)	19 (0.55)	1.0	
	Yes	544 (13.5)	6 (1.10)	1.5 (0.5–4.1)	
HIV testing	No	2735 (68.0)	11 (0.40)	1.0	1.0
	Other	590 (14.7)	12 (2.03)	3.0 (1.1–7.8)	4.0 (1.6–9.9)
	As part of antenatal care	696 (17.3)	2 (0.29)	1.1 (0.2–5.2)	1.3 (0.3–6.2)

^a^All variables based on status as at follow–up, except rural or urban residence and spouse HIV status. Total number of women in the socio-demographic only model was 4,207, in the behavioural only model was 3,752, and the combined model was 4,013 because of missing data for these variables: marital status for 85, education status for 85, occupation status for 87, Standard of living index for 12, sex with men for 549, had tattooing for 112, had blood transfusion for 112, smoking or chewing tobacco for 108, HIV testing other than ANC for 283, injections received in the last 12 months for 111, contacted by any one for HIV prevention programme for 281, travel outside place of residence for 89.

^b^Adjusted odds based on separate multiple logistic regression models for socio-demographic variables and for behavioural variables.

^c^Adjusted odds based on a single multiple logistic regression model that included socio-demographic and behavioural variables that were significant in the separate models.

^d^Standard of living index based on living conditions and ownership of assets, as used in National Family Health Survey-2 [27]; quartiles defined according to the baseline study distribution [18].

Though not statistically significant, the point estimates of odds of acquiring HIV were higher among men who relocated from rural to urban residence (3.9, 95% CI 0.7-20.5) and among women who relocated from urban to rural residence (2.4, 95% CI 0.3-20.4).

## Discussion

Assessment of seroconversion in a population cohort followed up over a period of time is the ideal method for estimation of HIV incidence. However, this also poses challenges related to loss to follow-up from migration and death, and the need for large samples in populations that do not have a high rate of HIV. These challenges were faced in our study, and we attempted to address the potential biases arising from these. We made extensive attempts to trace baseline study participants who had migrated to other parts of the district in order to include them in the follow-up study. Of the 12,066 eligible HIV negative persons from the baseline, we could reach a participation rate of 70% over 5–6 years of follow-up, higher for the rural (75%) than for the urban (64%) sample. We assessed the compositional bias due to the distribution of risk factors associated with HIV among those who participated in the follow-up study and those who did not, and adjusted for the modest estimated compositional bias due to differential participation. Though the approach to adjust for compositional bias may be debated, it may be preferable to do so when data are available regarding the distribution of risk variables among the participants and non-participants.

The striking finding from this first population-based cohort study of HIV incidence from India is that rural incidence in adults (1.47 per 1000 person-years) was about twice the urban incidence (0.70 per 1000 person-years), in the background of a not very different prevalence of HIV at baseline among rural (1.64%) and urban (1.89%) resident in Guntur district. Although the confidence intervals for the rural and urban incidence overlapped due to the relatively small number of HIV incidence cases (total 48), the two-fold difference is suggestive of a higher rural incidence. This trend was observed for both men and women. The crude HIV incidence rate in rural men was only 27% higher than in urban men (Table 4), but adjustment for the age and regional distribution of the Guntur district population resulted in an almost two-fold difference. In the multivariate models the rural–urban residence was not significantly associated with HIV incidence after adjusting for the other risk variables, suggesting that the distribution of risk variables for acquiring HIV was responsible for the higher incidence observed in the rural sample. For example, 89.6% of rural men were not circumcised versus 71.4% urban men, and 41.7% of rural men had more than one lifetime sex partner or had been to a sex worker versus 35.9% urban men. Among women the only identified risk factor that was more common among rural than urban women was tobacco use, 4.6% and 2.7%, respectively, suggesting that unidentified risk factors are contributing to the higher HIV incidence among rural women; related to this only 24% of the incidence variability among women was explained by the variables used in the multivariate model. Unidentified risk factors are also contributing to the higher HIV incidence among rural men in addition to the identified risk factors, as only 34% of the incidence variability was explained by the variables used in the multivariate model. While the HIV incidence trends found in Guntur district cannot be assumed to be applicable to all other parts of India, it is plausible that this trend may apply to some or a large proportion of districts that have a relatively high level of HIV as in Guntur. By comparison, a previous study from South Africa reported that between 2003 and 2005 the HIV incidence was higher among the urban than the rural population [3]. The relatively higher rural HIV incidence in our study, starting from a not very different HIV prevalence in rural and urban residents, indicates that there may be different trends of HIV in the rural and urban populations during the study period.

The incidence per 100 person-years was 3.7% of the baseline prevalence of HIV in urban adults and 9% in rural adults. This indicates that the assumption used sometimes that incidence per 100 person-years would be about 10% of the HIV prevalence may not apply in all Indian settings, and therefore incidence estimates based on this assumption may be erroneous.

The associations with HIV incidence that were found in this study could be a useful guide for focussing prevention efforts against new HIV infections. The strongest association of HIV incidence was with an HIV-positive spouse at baseline. Although the total number of persons with HIV-positive spouse in the population was small, the very strong odds of new HIV infection with this indicate the need for particular attention to HIV prevention efforts among couples in which one partner is HIV positive. Positive prevention and couple HIV testing and counselling for sero-discordant couples are part of the guidelines for HIV prevention interventions in India in general. However, the findings of this study indicate that more emphasis is needed on this prevention effort.

The other significant associations for HIV incidence among men were frequent use of condom for sex over the past 6 months, no circumcision, more than one life time woman sex partner or ever visited sex worker, and transport-related occupation. It is interesting to note that the first two of these were also significantly associated with HIV prevalence at baseline [25]. The positive association of HIV incidence with frequent condom use over the past 6 months is likely because men who got infected in the 5.63 years period between the baseline and follow-up visits may have adopted condom use as a result of their known infection status or perceived risk. Therefore, this association cannot be a causal. Among women, the other significant associations with HIV incidence were having had HIV testing other than for antenatal check-up, having been married previously but not currently, and tobacco smoking or chewing. The positive association of HIV incidence with HIV testing other than antenatal check-up and tobacco use among women cannot be causal, but is indicative of other hidden risk variables among them that could lead to higher risk of acquiring HIV infection. The association with previously but not currently married status may indicate that the women who acquire HIV are more likely to get divorced, or this marital status puts them at higher risk of acquiring HIV, or they may have acquired HIV from their spouse who died from HIV/AIDS.

The overall incidence was similar in those who had relocated between the baseline and follow-up studies and those who did not. Interestingly, the point estimates for the odds of acquiring HIV were 3.9 times higher for men who relocated from rural to urban residence versus all other men, and 2.4 times higher women who relocated from urban to rural residence versus all other women, though these were not statistically significant. Our study was not designed specifically to estimate the differences in HIV incidence among the various relocation groups, and therefore the lack of significant difference could potentially have been due to less power to assess this difference.

A significant limitation of our analysis is that we cannot comment on whether there was decreasing or increasing trend of incidence over the relatively long follow-up period averaging 5.63 years because we are not able to estimate the timing of having acquired HIV during this period. On the other hand, this relatively long follow-up period enabled a reasonable number of new HIV cases in the study that were needed for reliable estimation of incidence and assessment of its associations. Another point to consider while interpreting the results is that we had 30% loss to follow-up for estimating incidence. We assessed the composition of the baseline and follow-up and tried to adjust for the potential compositional bias, although the magnitude of this adjustment ended up being small. Comparison with the participation rates of previous longitudinal studies in sub-Saharan Africa reveals that the participation rate in our study was quite reasonable [1-12]. The follow-up rate in our study was lower for the urban than for rural participants. We addressed this in the incidence calculations by adjusting for potential compositional bias separately for urban and rural participants. Another limitation to consider is that although we interviewed the respondents in private settings and probed gently regarding sexual behaviour, it is possible that socially desirable behaviours could have been over-reported, which could have biased to some extent our assessment of associations with HIV incidence.

This study highlights that it can be challenging to do a population-based cohort study of HIV that would yield a large number of new HIV cases in India, unlike sub-Saharan African countries where the burden of HIV and the rate of new HIV infections is generally about an order of magnitude higher [1-12]. Nevertheless, these first direct HIV incidence data from India describing its distribution and associations in the population from a longitudinal study provide useful insights for strengthening HIV prevention services.

## Conclusions

These first population-based cohort incidence data from India reveal somewhat higher incidence in rural residents than in urban residents of a relatively high HIV-burden district, suggesting that rural areas of high HIV burden states may need more attention for prevention of new HIV infections. With the strongest association of HIV incidence found with having a HIV positive spouse, positive prevention needs to be implemented more effectively. In addition, the other associations found with acquiring new HIV infection indicate that these risk groups need to be targeted better by HIV prevention programmes.

## Competing interests

The authors declare that they have no competing interests.

## Authors’ contributions

LD and RD led the overall design, data collection, analysis and interpretation. GAK did the statistical analysis and contributed to interpretation. LD and GAK drafted the manuscript. VL led the laboratory analysis and interpretation. GMMA, MAkbar and SPR supervised the field data collection and contributed to interpretation of findings. TS contributed to the laboratory analysis and interpretation. MAlary contributed to the design and interpretation of findings. All authors approved the final version of the manuscript.

## Pre-publication history

The pre-publication history for this paper can be accessed here:

http://www.biomedcentral.com/1471-2334/13/327/prepub
